# The association of dietary choline and betaine and anthropometric measurements among Iranian children: a cross-sectional study

**DOI:** 10.1186/s12887-021-02677-1

**Published:** 2021-04-30

**Authors:** Alireza Jafari, Yahya Jalilpiran, Katherine Suitor, Nick Bellissimo, Leila Azadbakht

**Affiliations:** 1 Department of Community Nutrition, School of Nutritional Sciences and Dietetics, Tehran University of Medical Sciences, PO Box: 1416643931, Tehran, Iran; 2 Nutritional Health Team (NHT), Universal Scientific Education and Research Network (USERN), Tehran, Iran; 3 Students’ Scientific Research Center (SSRC), Tehran University of Medical Sciences (TUMS), Tehran, Iran; 4 School of Nutrition, Ryerson University, Toronto, Canada; 5 Diabetes Research Center, Endocrinology and Metabolism Clinical Sciences Institute, Tehran University of Medical Sciences, Tehran, Iran; 6 Department of Community Nutrition, School of Nutrition and Food Science, Isfahan University of Medical Science, Isfahan, Iran

**Keywords:** Choline, Betaine, Obesity, Overweight, Wasting, Underweight children

## Abstract

**Background:**

Previous studies have suggested that choline and betaine are associated with improved anthropometric measures including, BMI and waist circumference however, results are largely inconsistent and limited studies exist in children. Our objective was to investigate the relationship between dietary choline and betaine, and anthropometric measurements among Iranian children.

**Methods:**

In this cross-sectional study, dietary information was collected for 788 six-year-old children, who attended Tehran health centers in 2018. We measured dietary intakes using a valid and reliable semi-quantitative food frequency questionnaire. The USDA database was used to calculate dietary choline and betaine. We assessed anthropometric characteristics, physical activity, and socio-demographic status based on a reliable and valid protocol. Logistic regression adjusted for energy, physical activity, socio-economic status, and maternal age, physical activity, BMI, and HEI2015 was used to assess this association.

**Results:**

Free choline, glycero-phospho-choline, phospho-choline, phosphatidyl-choline, total choline, and total betaine, and choline were not related to overweight, obesity, underweight and wasting in the crude and adjusted model after controlling for children’s energy intake, children’s physical activity, socio-economic status, maternal physical activity, and BMI. Betaine intake was associated with mid-arm circumference and risk of overweight.

**Conclusions:**

We did not find any evidence to support the association between dietary choline with anthropometric measurements among Iranian children. Further prospective studies with a large sample size in different populations are needed.

## Background

Epidemiological studies suggest that well-nourished children have healthier growth and development patterns, which may lead to a lower risk of chronic disease later in life [1]. Still, childhood overweight and obesity have become significant public health issues [2]. The number of overweight or obese infants and young children (aged 0 to 5 years) increased from 32 million globally in 1990 to 41 million in 2016 [3]. In Iran, reports have shown that childhood obesity has risen from 5% to 15% between 2000 to 2018 [4]. Furthermore, based on a national study, six-year-old girls are at double the risk of being categorized as overweight compared to boys in Iran [5]. Childhood overweight is associated with an increased risk for obesity, insulin resistance, diabetes, dyslipidemia, fatty liver disease, and cardiovascular diseases in adulthood [6]. According to previous studies, various factors impact childhood obesity, such as diet [7, 8], physical activity, and socio-economic status (SES) [9, 10]. Notably, a double burden exists which suggests children who experience underweight or wasting are similarly at a higher risk for developing non-communicable diseases later in life, or suffering from undernutrition or nutrition deficiency, which can also lead to the development of infectious disease [11]. On a global scale, approximately 16% of children are considered underweight, and 9% are considered wasted [12]. In Iran, poor nutrition status impacted approximately 15.5% of children in 2017, with higher rates observed among girls [13, 14]. Interestingly, a meta-analysis showed a distinct parent-child resemblance in anthropometric measurements [15] and a different umbrella review reported that family-based interventions were helpful for obesity and weight management [16]. Together, this suggests that maternal influence and characteristics are important considerations for weight management programs and research.

Several studies have explored the association between individual foods, nutrients [17–19], and dietary patterns [20–23] and anthropometric measurements in children. However, there is limited evidence regarding the association between other dietary components such as choline and betaine, as methyl donor compounds, and anthropometric measurements. Choline and betaine are quaternary ammonium compounds synthesized from diet or de novo synthesis in tissues [24, 25]. Choline is the primary source of methyl groups in the diet, and is found in dietary sources including eggs, beef, liver, seafood, and milk [25]. Various esterified forms of choline exist with different metabolic impacts, bioavailability, and dietary sources, including phospho-choline, glycero-phospho-choline, and phosphatidyl-choline [26]. For example, phosphatidyl-choline has a role in gene expression, glucose homeostasis, and membrane structure, phospho-choline is the main form of choline in human breast milk and modulates immune function, and glycero-phospho-choline maintains osmotic regulation [27–29]. Betaine is abundant in marine invertebrates, wheat germ, spinach, and sugar beets [24]. Betaine supplementation has been shown to increase muscle mass, decrease body fat percentage and fat mass [30, 31]. Previous work in adults has shown that supplementation of dietary choline and betaine led to decreased visceral fat accumulation, waist circumference, and body weight which suggests they may contribute to the development of chronic disease [32, 33]. However, limited studies exist in children investigating their role, and findings in adults remain inconsistent [32, 34–36]. Children get ready to enter the school environment at six years of age and experience more independence to self-select and manage their own food menu choices [37]. Various esterified forms of choline have different metabolic impacts, bioavailability, and dietary sources, and no other study has investigated their role. Taken together with the higher rates of underweight and wasting observed in girls, it is critical to delineate the role that diet plays in the development of chronic disease in this age category. Therefore, we aimed to investigate the association between dietary choline forms and betaine and anthromopometric measures among Irainian six-year-old girls. 

## Methods and materials

### Study population

This cross-sectional study was performed on 788 children who attended Tehran health centers with their mothers for preschool vaccination from October 2017 to March 2018. Obesity and overweight prevalence was used to calculate sample size [5], on P_1_ = 0.22, P_2_ = 0.32, β = 0.2, α = 0.05 with the following formula: $n=\frac{{\left[\left({z}_{1-\frac{\alpha }{2}}\right)+\left({z}_{1-\beta}\right)\right]}^2\times \left({p}_1\left(1-{p}_1\right)+{p}_2\left(1-{p}_2\right)\right)}{{\left({p}_2-{p}_1\right)}^2}$. We interviewed 788 six-year-old girls to account for missing data. Participants were selected using a cluster random sampling method from 10 health and treatment centers. Parents provided written and informed consent for their child’s participation. Inclusion criteria information was collected from parents and included six-year-old girls without any history of acute or chronic disease, or specific diet. Participants were excluded in the final analysis stage if their estimated energy intake (calculated using NUTRITIONIST 4; version 7.0; NSquared Computing, Salem, OR) was not between 235 and 1735 kcal/d [38] (*n* = 0).

### Dietary intake assessment and choline calculation

Participants dietary intake was assessed using a reliable semi-quantitative food frequency questionnaire (FFQ) with 168 food items over the past year; validated in children [39, 40]. Dietary intakes were recorded based on face-to-face interviews with participant mothers. The FFQ collected the number of daily (e.g. bread), weekly (e.g. rice), or monthly (e.g. fish) standard servings using household measures of common food items consumed in Iran in the previous 12 months by a trained nutritionist. Reported food intakes were converted into grams per day (g/d), and entered into the NUTRITIONIST 4 (version 7.0; NSquared Computing, Salem, OR) software, modified for Iranian food items, and total calorie (kcal/day) and nutrient intakes were computed for each food item. Choline, glycero-phospho-choline, phospho-choline, phosphatidyl-choline, and betaine were calculated by multiplying each food item based on the United States Department of Agriculture (USDA) food content databases [41–45]. Total choline intake was calculated as the sum of choline intake from free choline, glycero-phospho-choline, phospho-choline, and phosphatidyl-choline. The sum of total choline and betaine together was used to calculate total betaine and choline.

### Anthropometric, socio-demographic, and physical activity measurement

Subjects were weighed (to the nearest 0.1 kg) with minimum coverage using an electronic scale (Seca 753E; Seca Weighing and Measuring Systems, Hamburg, Germany). Standing height was measured (to the nearest 0.1 cm) in a fixed position without shoes, and body mass index (BMI) was calculated (kg/m^2^). Mid-arm circumference was measured based on the length between the mid-point of the elbow and the shoulder (at 0.1 mm) precision. World Health Organization (WHO) diagnostic criteria was used to diagnose overweight or obesity in children: obese (BMI-for-age ≥ 2SD), overweight (BMI-for-age ≥ 1SD), normal (BMI-for-age ≥ −1SD and < 1SD), underweight (BMI-for-age < −1SD) and wasting (BMI-for-age < −2SD) [46]. Physical activity levels were recorded based on self-reported measures by the mothers of participants based on the average time of indoor and outdoor physical activity over a 24 h period, according to light, moderate, high, and very high intensity. These values were multiplied by their corresponding metabolic equivalent, stated as metabolic equivalent hours per week (MET h/wk) [47], and total physical activity was calculated using the International Physical Activity Questionnaire. We categorized participants socioeconomic status (SES) according to a validated Persian questionnaire, which included questions related to the information about the parent’s occupation and education, number of children under 18 years, number of family members, income level, health care insurance, food assistance, dwelling ownership, neighborhood, vehicle ownership, number of cars and number of rooms in the house [48]. A standardized SES score was calculated using factor analysis and a summary index, and compliance using a Kappa test. The correlation of these parameters with the standardized SES score was reported at 0.87.

### Statistical analysis

A Kolmogorov-Smirnov test and histogram curve was used to test the normality of quantitative variables. A one-way analysis of variance (ANOVA) was used to compare quantitative variables across total dietary to choline, total betaine, and total choline and betaine tertiles based on population distribution. An analysis of covariance (ANCOVA) adjusted for daily energy intake using the residual method and Healthy Eating Index (HEI) was used to compare dietary intake of macro and micronutrients across tertiles of total choline, total betaine, and total choline and betaine [49]. Statistical interaction between dietary choline, glycero-phospho-choline, phospho-choline, phosphatidyl choline, total choline, total betaine, and total betaine and choline with participant height-for-age and mid-arm circumference was analyzed using linear regression. Multivariable binary logistic regressions were used to assess the association between participants’ anthropometric measurements and dietary choline, glycero-phospho-choline, phospho-choline, phosphatidyl-choline, total choline, total betaine, and total betaine and choline. Participant energy intake, physical activity, SES, maternal age, physical activity, BMI, and Healthy Eating Index 2015 score (HEI2015) were adjusted for. Partial correlation analysis controlling for energy and HEI was used to evaluate the correlation of dietary choline, glycero-phospho-choline, phospho-choline, phosphatidyl choline, total choline, total betaine, and total betaine and choline with participants characteristics. Statistical analyses were performed using SPSS software (version 26, SPSS Inc., Chicago, IL, USA). A *P* < 0.05 was considered statistically significant.

### Ethical consideration

The National Institute for Medical Research Development of Tehran University of Medical Science, Tehran, Iran (IR. TUMS. VCR.REC.94–04–161-31,112) was granted ethical approval from the Ethics Commission of Tehran University of Medical Science. Verbal and written informed consent was obtained from parents for the participation of their children in the study.

## Results

Demographic characteristics of the 788 participants and mothers are shown in Table 1. All participants remained in the study for the entire study duration. The mean age, BMI, physical activity, and energy intake of mothers was: 32 years, 24.88, 30 MET/h, and 2267 kcal. Participants mean intake of free choline (57 mg/day), glycerol-phospho-choline (54 mg/day), phospho-choline (12 mg/day), phosphatidyl choline (140 mg/day), total choline (263 mg/day), total betaine (71 mg/day), and total choline and betaine (334 mg/day) are reported in Table 1.

**Table 1:** Participant characteristics (*n* = 788)

Variables	Maternal	Participants
Age (Years)	32.06 ± 8.58	–
BMI (kg/m^2^)	24.88 ± 4.53	–
Physical activity (MET/h)	30.09 ± 3.82	9.50 ± 0.36
Total Energy intake (Kcal/day)	2267.33 ± 733.84	1014.75 ± 259.16
Body mass index-for-age (BAZ)	–	0.37 ± 1.09
Height-for-age (HAZ)	–	−0.55 ± 0.42
Mid arm circumference (cm)	–	15.46 ± 0.54
SES	–	31.87 ± 6.81
Free Choline (mg/day)	–	57 ± 27
Glycero-phospho-choline (mg/day)	–	54 ± 26
Phospho-choline (mg/day)	–	12 ± 5
Phosphatidyl choline (mg/day)	–	140 ± 90
Total choline (mg/day)	–	263 ± 137
Betaine (mg/day)	–	71 ± 33
Total choline and betaine (mg/day)	–	334 ± 151

Data are presented Mean ± SD

*BMI* body mass index; *SD* standard deviation; *SES* socioeconomic status

Table 2 represents general characteristics of participants across dietary total choline, betaine, and total choline and betaine tertiles. Accordingly, participants with a greater total betaine intake had lower energy intake (985 kcal, *P* = 0.013) and estimated SES (32, *P* = 0.035).

**Table 2:** Participants characteristics across total choline, betaine, and total choline and betaine intake tertiles (n = 788)

Variables	Total choline				Total betaine				Total choline and betaine			
	T1(*n* = 262)	T2(*n* = 263)	T3(n = 263)	*P	T1(n = 263)	T2(n = 263)	T3(n = 262)	*P	T1(n = 262)	T2(n = 263)	T3(n = 263)	*P
**Maternal age (Year)**	32.13 (9.04)	31.76 (8.21)	31.77 (8.52)	0.684	32 (8.59)	32.15 (8.88)	31.51 (8.3)	0.555	32 (8.93)	31.91 (8.36)	31.74 (8.49)	0.819
**Maternal BMI (kg/m2)**	24.9 (4.61)	24.85 (4.53)	24.54 (4.14)	0.628	24.75 (4.85)	25.17 (4.48)	24.38 (4.17)	0.388	24.82 (4.69)	24.94 (4.51)	24.54 (4.34)	0.414
**Maternal physical activity (MET/h)**	29.83 (3.62)	30.14 (4.21)	30.13 (3.87)	0.116	30.14 (3.8)	30.13 (3.96)	29.82 (3.96)	0.386	29.99 (3.73)	30.02 (4.09)	30.08 (3.91)	0.194
**Participants physical activity (MET/h)**	9.52 (0.37)	9.49 (0.35)	9.48 (0.35)	0.308	9.50 (0.37)	9.50 (0.34)	9.49 (0.36)	0.925	9.50 (0.37)	9.49 (0.35)	9.50 (0.35)	0.876
**Participants total energy intake (Kcal/day)**	987.94 (240.45)	1062.40 (266.31)	993.55 (264.16)	0.803	1084.19 (235.42)	975.46 (265.77)	984.86 (262.00)	0.013	1003.46 (254.92)	1051.21 (257.58)	989.53 (261.86)	0.537
**BMI-for-age (BAZ)**	0.31 (1.13)	0.38 (1.06)	0.41 (1.09)	0.334	0.23 (1.15)	0.48 (1.01)	0.38 (1.11)	0.129	0.34 (1.15)	0.34 (1.04)	0.41 (1.09)	0.452
**Height-for-age HAZ)**	−0.58 (0.42)	−0.52 (0.42)	−0.56 (0.44)	0.673	−0.54 (0.40)	−0.54 (0.44)	−0.58 (0.43)	0.297	−0.57 (0.41)	−0.54 (0.43)	− 0.56 (0.43)	0.777
**Mid-arm circumference(cm)**	15.44 (0.53)	15.47 (0.59)	15.46 (0.49)	0.679	15.41 (0.54)	15.46 (0.54)	15.49 (0.53)	0.059	15.42 (0.55)	15.46 (0.56)	15.49 (0.49)	0.152
**Socioeconomic status (SES)**	32.28 (6.88)	32.07 (6.93)	32.25 (6.60)	0.085	32.16 (7.07)	32.28 (6.75)	31.87 (6.81)	0.035	32.25(6.88)	32.19(7.16)	31.16(6.34)	0.066

*BMI* body mass index; *SD* standard deviation; *T* tertile

Data presented as mean (SD)

* Denotes *P* values calculated using ANOVA test. P < 0.05 was considered significant

Energy and HEI2015 adjusted dietary intakes across tertiles of dietary choline and betaine intakes are shown in Table 3. Accordingly, participants with a higher intake of total choline and betaine had a greater intake of macronutrients and micronutrients (*P* < 0.05) however this significant association was not observed for vitamin E and total fiber.

**Table 3:** Energy adjusted dietary intakes and tertiles of choline and betaine intake among children (n = 788)

Dietary component intake	Total choline				Total betaine				Total choline and betaine			
	T1 (n = 262)	T2 (n = 263)	T3 (n = 263)	*P	T1 (n = 262)	T2 (n = 263)	T3 (n = 263)	*P	T1 (n = 262)	T2 (n = 263)	T3 (n = 263)	*P
**Protein (g/day)**	32.24 (0.61)	41.41 (5.34)	44.57 (1.00)	0.02	35.29 (0.69)	34.93 (0.83)	48.01 (5.35)	0.005	31.73 (0.61)	36.84 (0.78)	49.65 (5.35)	< 0.001
**Fat (g/day)**	29.03 (0.63)	32 (0.67)	40.43 (0.98)	< 0.001	31.91 (0.67)	30.93 (0.74)	38.63 (0.97)	< 0.001	28.91 (0.63)	32.09 (0.67)	40.46 (0.98)	< 0.001
**CHO (g/day)**	147.4 (2.62)	148.93 (3.06)	165.51 (4.06)	< 0.001	150.4 (2.90)	144.54 (3.02)	165.92 (3.89)	< 0.001	144.68 (2.70)	152.9 (3.06)	163.25 (4.02)	< 0.001
**Total Fiber (g/day)**	5.65 (0.17)	6.18 (0.28)	7.56 (0.26)	< 0.001	6.53 (0.29)	6 (0.25)	6.85 (0.19)	0.196	5.53 (0.16)	6.35 (0.28)	7.51 (0.26)	< 0.001
**SFA (mg/day)**	17.82 (0.44)	21.7 (0.49)	27.97 (0.74)	< 0.001	21.47 (0.55)	20.24 (0.57)	25.78 (0.71)	< 0.001	18.05 (0.46)	21.43 (0.49)	28.01 (0.73)	< 0.001
**Iron (mg/day)**	15.57 (0.27)	15.87 (0.30)	17.29 (0.33)	< 0.001	15.76 (0.27)	15.23 (0.30)	17.74 (0.32)	< 0.001	15.25 (0.28)	16.02 (0.29)	17.45 (0.33)	< 0.001
**Magnesium (mg/day)**	234.97 (4.81)	268.15 (6.76)	325.5 (7.78)	< 0.001	269.13 (6.60)	252.8 (6.80)	306.82 (7.06)	< 0.001	233.3 (4.95)	269.24 (6.65)	326.07 (7.74)	< 0.001
**Zinc (mg/day)**	6.97 (0.13)	8.74 (0.14)	9.64 (0.13)	< 0.001	8.19 (0.14)	7.98 (0.16)	9.18 (0.14)	< 0.001	6.94 (0.13)	8.77 (0.14)	9.63 (0.13)	< 0.001
**Potassium (mg/day)**	2853.93 (69.82)	3239.44 (84.40)	3047.47 (95.65)	< 0.001	3271.85 (84.00)	3052.24 (86.93)	3718.34 (89.54)	< 0.001	2825.69 (69.22)	3263.81 (83.66)	3951.24 (96.16)	< 0.001
**Calcium (mg/day)**	426.63 (9.90)	504.58 (10.62)	504.58 (11.92)	< 0.001	483.38 (10.71)	447.62 (10.58)	564.5 (11.63)	< 0.001	428.03 (10.19)	497.65 (10.32)	569.62 (11.86)	< 0.001
**Phosphorus (mg/day)**	517.08 (11.71)	623.1 (13.61)	768.91 (12.29)	< 0.001	581.15 (12.92)	576.35 (14.62)	751.47 (16.93)	< 0.001	508.06 (11.46)	630.14 (13.47)	770.86 (17.28)	< 0.001
**Vitamin B9 (μg/day)**	271.85 (7.34)	316.90 (11.24)	378.46 (12.00)	< 0.001	311.19 (10.85)	292.68 (10.03)	363.48 (10.86)	< 0.001	265.31 (7.24)	320.28 (10.97)	381.59 (12.14)	< 0.001
**Vitamin B12 (μg/day)**	2.67 (0.08)	3.95 (0.09)	4.71 (0.09)	< 0.001	3.47 (0.09)	3.48 (0.10)	4.37 (0.10)	< 0.001	2.74 (0.08)	3.85 (0.08)	4.74 (0.09)	< 0.001
**Vitamin A (RAE/day)**	1273.57 (79.00)	1293.32 (45.64)	1562.55 (55.09)	< 0.001	1353.31 (55.86)	1258.55 (67.60)	1517.89 (60.93)	0.032	1226.4 (73.75)	1331.41 (52.52)	1571.45 (55.82)	< 0.001
**Vitamin D (μg/day)**	0.99 (0.06)	1.65 (0.09)	2.31 (0.10)	< 0.001	1.51 (0.10)	1.43 (0.08)	2.01 (0.10)	< 0.001	0.97 (0.06)	1.67 (0.09)	2.31 (0.1)	< 0.001
**Vitamin K (μg /day)**	116.09 (4.07)	136.31 (5.41)	168.43 (6.51)	< 0.001	137.73 (5.51)	121.68 (5.07)	161.5 (5.88)	< 0.001	114.29 (3.93)	138.6 (5.74)	167.94 (6.30)	< 0.001
**Vitamin E (mg/day)**	11.10 (0.34)	11.69 (0.39)	12.09 (0.41)	0.086	11.92 (0.39)	10.78 (0.34)	12.2 (0.41)	0.068	11.11 (0.35)	11.72 (0.38)	12.05 (0.42)	0.136

*CHO* carbohydrate; *T* tertile; *SD* standard deviation; *SE* standard error; *SFA* saturated fatty acid

Data are presented as Mean ± SE (standard error)

* denotes P values calculated using ANCOVA

^1^ All the variables adjusted for energy intake and HEI2015

Crude and multivariable odds ratio (OR) and 95% confidence intervals (CI) for overweight, obesity, underweight, and wasting across free choline, glycero-phospho-choline, phospho-choline, phosphatidyl choline, total choline, total betaine, and total betaine and choline tertiles are shown in Table 4. After adjusting for potential confounders, including participant energy intake, physical activity, SES, maternal age, physical activity, BMI, and HEI2015, there was no significant association between total choline, and risk of wasting (OR: 1.00; 95% CI 0.35–2.89), obesity (OR: 0.94; 95% CI 0.33–2.71), overweight/obesity (OR: 1.08; 95% CI 0.74–1.56), and underweight/wasting (OR: 1.04; 95% CI 0.63–1.73). Participants in the lowest tertiles of total betaine intake were not associated with a higher risk of wasting (OR: 1.00; 95% CI 0.32–3.14), obesity (OR: 1.30; 95% CI 0.42–4.09), and underweight/wasting (OR: 0.90; 95% CI 0.53–1.51). Similarly, a higher intake of total choline and betaine was not associated with risk of wasting (OR: 1.05; 95% CI 0.36–3.03), obesity (OR: 0.77; 95% CI 0.28–2.13), overweight/obesity (OR: 1.01; 95% CI 0.69–1.47), and underweight/wasting (OR: 1.13; 95% CI 0.68–1.87). Participants in the highest dietary betaine intake group had a significantly higher risk of overweight/obesity (OR: 1.49; 95% CI 1.02–2.18).

**Table 4:** Risk for wasting, underweight, overweight, and obesity across participant tertiles of total choline, total betaine, and total choline and betaine intake in six-year-old Iranian girls (n = 788)

Outcomes	Free Choline				Glycero-phospho-choline				Phospho-choline				Phosphatidyl-choline			
	T1	T2	T3	†P	T1	T2	T3	†P	T1	T2	T3	†P	T1	T2	T3	†P
**Obesity (BMI for age z-score ≥ + 2 SD)**																
**Case/control**	9/253	7/256	7/256	–	4/258	12/252	7/255	–	7/255	9/254	7/256	–	11/251	6/257	6/257	–
**Crude**	1	0.77 (0.28–2.10)	0.77 (0.28–2.10)	0.790	1	3.07 (0.98–9.65)	1.77 (0.51–6.12)	0.483	1	1.29 (0.47–3.52)	1.00 (0.34–2.88)	0.994	1	0.53 (0.19–1.46)	0.53 (0.19–1.46)	0.196
**Model 1**	1	0.77 (0.28–2.10)	0.77 (0.28–2.11)	0.768	1	3.32 (1.05–10.48)	1.80 (0.52–6.23)	0.422	1	1.36 (0.50–3.72)	1.00 (0.34–2.89)	0.998	1	0.54 (0.20–1.47	0.53 (0.19–1.46)	0.197
**Model 2**	1	0.74 (0.27–2.03)	0.75 (0.28–2.07)	0.815	1	3.11 (0.98–9.87)	1.69 (0.49–5.90)	0.489	1	1.40 (0.51–3.83)	0.96 (0.33–2.80)	0.942	1	0.56 (0.20–1.55)	0.55 (0.20–1.53)	0.228
**Model 3**	1	0.76 (0.28–2.09)	0.81 (0.29–2.29)	0.912	1	3.16 (1.00–10.06)	1.79 (0.51–6.27)	0.429	1	1.43 (0.52–3.96)	1.03 (0.35–3.07)	0.950	1	0.57 (0.21–1.58)	0.58 (0.20–1.65)	0.271
**Overweight and obesity (BMI for age z score ≥ + 1 SD)**																
**Case/control**	82/180	91/172	84/179	–	80/182	93/171	84/178	–	83/179	88/175	86/177	–	77/185	89/174	91/172	–
**Crude**	1	1.16 (0.81–1.67)	1.03 (0.71–1.49)	0.534	1	1.24 (0.86–1.78)	1.07 (0.74–1.55)	0.709	1	1.08 (0.75–1.56)	1.05 (0.73–1.51)	0.803	1	1.23 (0.85–1.78)	1.27 (0.88–1.84)	0.203
**Model 1**	1	1.17 (0.81–1.68)	1.02 (0.71–1.48)	0.553	1	1.25 (0.86–1.80)	1.06 (0.73–1.54)	0.751	1	1.09 (0.76–1.58)	1.04 (0.72–1.50)	0.831	1	1.24 (0.86–1.79)	1.27 (0.88–1.83)	0.209
**Model 2**	1	1.17 (0.81–1.68)	1.02 (0.71–1.48)	0.553	1	1.24 (0.86–1.79)	1.06 (0.73–1.53)	0.779	1	1.09 (0.76–1.58)	1.03 (0.72–1.49)	0.860	1	1.25 (0.86–1.81)	1.27 (0.88–1.84)	0.205
**Model 3**	1	1.13 (0.79–1.64)	0.97 (0.67–1.41)	0.700	1	1.23 (0.85–1.77)	1.03 (0.71–1.49)	0.888	1	1.08 (0.75–1.56)	0.99 (0.68–1.44)	0.965	1	1.23 (0.85–1.78)	1.22 (0.84–1.78)	0.306
**Underweight and wasting (BMI for age z-score < −1 SD)**																
**Case/control**	34/228	35/228	36/227	–	33/229	31/233	41/221	–	35/227	34/229	36/227	–	36/226	33/230	36/227	–
**Crude**	1	1.03 (0.62–1.71)	1.06 (0.64–1.76)	0.986	1	0.92 (0.55–1.56)	1.29 (0.79–2.11)	0.304	1	0.96 (0.58–1.60)	1.03 (0.62–1.70)	0.911	1	0.90 (0.54–1.50)	1.00 (0.61–1.64)	0.986
**Model 1**	1	1.01 (0.61–1.68)	1.08 (0.65–1.80)	0.953	1	0.90 (0.53–1.52)	1.33 (0.81–2.19)	0.247	1	0.92 (0.55–1.54)	1.05 (0.63–1.73)	0.853	1	0.88 (0.53–1.47)	1.01 (0.61–1.66)	0.972
**Model 2**	1	1.01 (0.61–1.69)	1.09 (0.66–1.82)	0.917	1	0.90 (0.53–1.53)	1.35 (0.82–2.21)	0.235	1	0.93 (0.56–1.55)	1.06 (0.64–1.75)	0.824	1	0.88 (0.53–1.47)	1.02 (0.62–1.69)	0.993
**Model 3**	1	1.02 (0.61–1.71)	1.12 (0.67–1.87)	0.872	1	0.91 (0.53–1.54)	1.36 (0.82–2.24)	0.220	1	0.93 (0.56–1.56)	1.08 (0.64–1.79)	0.779	1	0.89 (0.53–1.49)	1.04 (0.63–1.73)	0.875
**Wasting (BMI for age z score < −2 SD)**																
**Case/control**	8/254	6/257	6/257	–	8/254	2/262	10/252	–	8/262	3/260	9/263	–	9/253	4/259	7/256	–
**Crude**	1	0.74 (0.25–2.17)	0.74 (0.25–2.17)	0.776	1	0.24 (0.05–1.15)	1.26 (0.49–3.24)	0.168	1	0.37 (0.10–1.40)	1.13 (0.43–2.96)	0.787	1	0.43 (0.13–1.43)	0.77 (0.28–2.10)	0.575
**Model 1**	1	0.74 (0.25–2.17)	0.72 (0.25–2.12)	0.729	1	0.25 (0.05–1.21)	1.22 (0.47–3.16)	0.159	1	0.38 (0.10–1.47)	1.09 (0.41–2.89)	0.835	1	0.44 (0.13–1.46)	0.76 (0.28–2.07)	0.558
**Model 2**	1	0.75 (0.26–2.20)	0.73 (0.25–2.15)	0.754	1	0.26 (0.06–1.23)	1.25 (0.48–3.23)	0.171	1	0.39 (0.10–1.48)	1.12 (0.42–2.97)	0.803	1	0.44 (0.13–1.47)	0.77 (0.28–2.11)	0.581
**Model 3**	1	0.82 (0.28–2.43)	0.87 (0.29–2.62)	0.981	1	0.26 (0.06–1.25)	1.41 (0.54–3.68)	0.281	1	0.41 (0.11–1.58)	1.34 (0.49–3.61)	0.578	1	0.46 (0.14–1.55)	0.93 (0.33–2.63)	0.832
**Outcomes**	**Total choline**					**Betaine**				**Total choline and betaine**						
	T1	T2	T3	†P	T1	T2	T3	†P	T1	T2	T3	†P				
**Obesity (BMI for age z-score ≥ + 2 SD)**																
**Case/control**	8/254	8/255	7/256	–	6/256	10/253	7/256	–	10/252	6/257	7/256	–				
**Crude**	1	1.00 (0.37–2.70)	0.74 (0.25–2.17)	0.124	1	1.69 (0.60–4.71)	0.62 (0.2–1.91)	0.801	1	0.59 (0.21–1.64)	0.69 (0.26–1.84)	0.434				
**Model 1**	1	0.99 (0.37–2.68)	0.86 (0.31–2.40)	0.152	1	1.62 (0.57–4.58)	1.17 (0.38–3.57)	0.804	1	0.61 (0.22–1.70)	0.69 (0.26–1.85)	0.442				
**Model 2**	1	1.02 (0.37–2.76)	0.88 (0.31–2.49)	0.180	1	1.68 (0.59–4.74)	1.19 (0.39–3.64)	0.777	1	0.62 (0.22–1.74)	0.73 (0.27–1.95)	0.449				
**Model 3**	1	1.04 (0.38–2.83)	0.94 (0.33–2.71)	0.216	1	1.77 (0.62–5.05)	1.30 (0.42–4.09)	0.663	1	0.64 (0.23–1.80)	0.77 (0.28–2.13)	0.581				
**Overweight and obesity (BMI for age z score ≥ + 1 SD)**																
**Case/control**	81/181	88/175	88/175	–	73/189	85/178	99/164	–	83/179	87/176	87/176	–				
**Crude**	1	1.12 (0.78–1.62)	1.12 (0.78–1.62)	0.514	1	1.24 (0.85–1.80)	1.56 (1.08–2.26)	0.017	1	1.07 (0.74–1.54)		0.732				
**Model 1**	1	1.13 (0.78–1.63)	1.12 (0.78–1.61)	0.520	1	1.23 (0.84–1.79)	1.55 (1.07–2.25)	0.020	1	1.07 (0.74–1.54)	1.06 (0.73–1.53)	0.759				
**Model 2**	1	1.14 (0.79–1.64)	1.12 (0.77–1.62)	0.502	1	1.23 (0.85–1.80)	1.55 (1.07–2.24)	0.021	1	1.07 (0.74–1.55)	1.06 (0.73–1.53)	0.763				
**Model 3**	1	1.12 (0.77–1.61)	1.08 (0.74–1.56)	0.720	1	1.21 (0.82–1.76)	1.49 (1.02–2.18)	0.038	1	1.05 (0.73–1.52)	1.01 (0.69–1.47)	0.961				
**Underweight and wasting (BMI for age z-score < −1 SD)**																
**Case/control**	36/226	33/230	36/227	–	37/225	36/227	32/231	–	36/226	31/232	38/225	–				
**Crude**	1	0.90 (0.54–1.50)	1.00 (0.61–1.64)	0.797	1	0.96 (0.59–1.58)	0.84 (0.51–1.40)	0.510	1	0.84 (0.50–1.40)	1.1 (0.67–1.8)	0.811				
**Model 1**	1	0.89 (0.53–1.38)	1.02 (0.62–1.68)	0.798	1	1.00 (0.61–1.65)	0.87 (0.52–1.45)	0.589	1	0.82 (0.49–1.38)	1.08 (0.66–1.77)	0.757				
**Model 2**	1	0.89 (0.53–1.48)	1.03 (0.62–1.70)	0.783	1	1.00 (0.61–1.65)	0.88 (0.53–1.47)	0.629	1	0.83 (0.50–1.39)	1.1 (0.67–1.81)	0.706				
**Model 3**	1	0.89 (0.54–1.49)	1.04 (0.63–1.73)	0.705	1	1.01 (0.61–1.68)	0.90 (0.53–1.51)	0.681	1	0.84 (0.50–1.41)	1.13 (0.68–1.87)	0.650				
**Wasting (BMI for age z score < −2 SD)**																
**Case/control**	8/254	5/258	7/256	–	7/255	7/256	6/257	–	8/254	5/258	7/256	–				
**Crude**	1	0.62 (0.20–1.91)	0.87 (0.31–2.43)	0.781	1	1.00 (0.34–2.88)	0.85 (0.28–2.57)	0.776	1	0.62 (0.20–1.91)	0.87 (0.31–2.43)	0.776				
**Model 1**	1	0.61 (0.20–1.90)	0.87 (0.31–2.44)	0.788	1	0.91 (0.31–2.67)	0.80 (0.26–2.44)	0.692	1	0.63 (0.20–1.94)	0.85 (0.30–2.38)	0.743				
**Model 2**	1	0.61 (0.20–1.91)	0.85 (0.30–2.40)	0.802	1	0.91 (0.31–2.69)	0.82 (0.27–2.50)	0.722	1	0.64 (0.20–1.99)	0.87 (0.31–2.45)	0.778				
**Model 3**	1	0.65 (0.21–2.03)	1.00 (0.35–2.89)	0.845	1	1.04 (0.35–3.07)	1.00 (0.32–3.14)	0.998	1	0.68 (0.22–2.15)	1.05 (0.36–3.03)	0.958				

Data presented as odds ratio and 95% confidence interval

*BMI* body mass index; *SD* standard deviation; *T* tertile

Crude: Not adjusted for any variables

Model 1: The model was adjusted for participants energy intake, physical activity, and SES

Model 2: The model was adjusted for participants energy intake, physical activity, SES, maternal age, BMI, and physical activity

Model 3: The model was adjusted for participants energy intake, physical activity, SES, maternal age, BMI, physical activity, and HEI2015

† P values are Calculated using multivariable binary logistic regression

P < 0.05 considered statistically significant

Multiple linear regression was used to assess the potential relationship between free choline, glycero-l phospho-choline, phospho-choline, phosphatidyl choline, total choline, total betaine, and total betaine and choline, and height-for-age, and mid-arm circumference, adjusted for participant energy intake, physical activity, SES (Model 1), and maternal age, BMI, physical activity, and HEI2015 (Model 2, Table 5). Total betaine intake was positively associated with mid-arm circumference (β = 0.087, *P* = 0.018). There was no significant relationship between free choline, glycero-phospho-choline, phospho-choline, phosphatidyl choline, total choline, and total choline and betaine.

**Table 5:** Association between height-for-age and mid-arm circumference and dietary free choline, glycero-phospho-choline, phospho-choline, phosphatidyl choline, total choline, betaine, and total choline and betaine intake among six-year-old Iranian girls (n = 788)

Variable	Free choline			Glycero-phospho-choline			Phospho-choline			Phosphatidyl-choline			Total choline			Betaine			Total choline and betaine		
	β	SE	P	β	SE	P	β	SE	P	β	SE	P	β	SE	P	β	SE	P	β	SE	P
**Height-for-age (HAZ)**																					
**Crude**	−0.028	0.019	0.429	− 0.005	0.019	0.885	0.008	0.019	0.817	0.004	0.019	0.921	0.002	0.019	0.963	−0.06	0.019	0.092	−0.024	0.019	0.493
**Model 1**	−0.029	0.019	0.415	− 0.007	0.019	0.850	0.007	0.019	0.837	0.003	0.019	0.931	0	0.019	0.997	−0.067	0.019	0.064	−0.026	0.019	0.468
**Model 2**	−0.027	0.019	0.447	−0.005	0.019	0.885	0.010	0.019	0.780	0.007	0.019	0.846	0.004	0.019	0.922	−0.062	0.019	0.084	−0.021	0.019	0.553
**Model 3**	−0.029	0.019	0.427	−0.006	0.019	0.875	0.010	0.019	0.792	0.006	0.019	0.861	0.003	0.019	0.935	−0.066	0.019	0.074	−0.023	0.019	0.532
**Mid-arm circumference (cm)**																					
**Crude**	0.059	0.023	0.098	0.034	0.024	0.346	0.026	0.024	0.470	0.044	0.023	0.222	0.054	0.023	0.127	0.083	0.023	0.020	0.043	0.023	0.225
**Model 1**	0.058	0.023	0.105	0.032	0.024	0.374	0.024	0.023	0.494	0.043	0.023	0.232	0.051	0.023	0.149	0.076	0.024	0.035	0.041	0.024	0.248
**Model 2**	0.058	0.023	0.105	0.030	0.024	0.398	0.023	0.023	0.524	0.047	0.024	0.184	0.054	0.023	0.127	0.077	0.024	0.033	0.045	0.024	0.207
**Model 3**	0.066	0.024	0.068	0.034	0.024	0.344	0.029	0.024	0.425	0.056	0.024	0.125	0.061	0.024	0.091	0.087	0.024	0.018	0.024	0.024	0.144

β denotes standardized coefficients obtained from Linear regression; SE denotes standard error

Crude: Not adjusted for any variables

Model 1: The model was adjusted for energy, physical activity, and SES

Model 2: The model was adjusted for energy, physical activity, SES, age, BMI, and physical activity

Model 3: The model was adjusted for energy, physical activity, SES, age, BMI, physical activity, and HEI2015

P value < 0.05 was considered significant

Table 6 demonstrates the adjusted Pearson correlations between participants’ characteristics and dietary free choline, glycero-phospho-choline, phospho-choline, phosphatidyl choline, total choline, total betaine, and total choline and betaine intake among participants. There was a correlation between the mid-arm circumference (r = 0.093, *P* = 0.009) and age (r = − 0.072, *P* = 0.044) with total betaine intake. With the exception of vitamin E, the dietary intake of children was correlated with free choline, glycero-phospho-choline, phospho-choline, phosphatidyl choline, total choline, and total betaine and choline. There was no significant correlation observed between dietary free choline, glycero-phospho-choline, phospho-choline, phosphatidyl choline, total choline, and total choline and betaine intake, and maternal age, BMI, physical activity, participant physical activity, BMI-for-age, height-for-age, or mid-arm circumference.

**Table 6:** Correlation between participant characteristics and dietary free choline, glycero-phospho-choline, phospho-choline, phosphatidyl choline, total choline, total betaine, and total choline and betaine intake among six-year-old Iranian girls (n = 788)

Variable	Free Choline		Glycero-phospho-choline		Phospho-choline		Phosphatidyl-choline		Total choline		Betaine		Total choline and betaine	
	R	P	R	P	R	P	R	P	R	P	R	P	R	P
**Maternal age (Year)**	−0.350	0.326	−0.051	0.155	−0.044	0.217	0.001	0.983	−0.018	0.620	−0.072	0.044	−0.032	0.377
**Maternal BMI (kg/m**^**2**^**)**	−0.014	0.685	−0.030	0.400	−0.031	0.383	−0.014	0.704	−0.019	0.601	−0.062	0.084	−0.030	0.397
**Participants BMI (kg/m**^**2**^**)**	0.044	0.222	0.034	0.347	0.025	0.476	0.046	0.196	0.046	0.194	0.017	0.624	0.046	0.196
**Maternal physical activity (MET/h)**	−0.013	0.717	−0.014	0.702	−0.011	0.753	0.020	0.582	0.007	0.836	0.005	0.892	0.008	0.827
**Participant physical activity (MET/h)**	−0.027	0.456	−0.047	0.191	−0.046	0.196	−0.029	0.417	−0.027	0.492	−0.029	0.416	−0.038	0.285
**BMI-for-age (BAZ)**	0.070	0.05	0.041	0.249	0.039	0.278	0.053	0.138	0.058	0.104	0.021	0.558	0.058	0.107
**Height-for-age (HAZ)**	−0.017	0.639	−0.002	0.954	0.002	0.965	0.037	0.298	0.021	0.560	−0.064	0.071	0.005	0.882
**Mid-arm circumference(cm)**	0.069	0.053	0.025	0.483	0.038	0.282	0.048	0.171	0.051	0.150	0.093	0.009	0.067	0.061
**SES**	−0.026	0.472	−0.031	0.387	−0.041	0.252	−0.036	0.311	−0.036	0.309	−0.042	0.242	−0.042	0.238
**Protein (g/day)**	0.087	0.014	0.086	0.016	0.096	0.007	0.091	0.011	0.097	0.006	0.28	< 0.001	0.177	< 0.001
**Fat (g/day)**	0.411	< 0.001	0.319	< 0.001	0.406	< 0.001	0.341	< 0.001	0.392	< 0.001	0.302	< 0.001	0.39	< 0.001
**CHO (g/day)**	0.159	< 0.001	0.112	0.002	0.146	< 0.001	0.093	0.009	0.120	0.001	0.248	< 0.001	0.248	< 0.001
**Total Fiber (g/day)**	0.188	< 0.001	0.129	< 0.001	0.195	< 0.001	0.175	< 0.001	0.184	< 0.001	0.192	< 0.001	0.267	< 0.001
**SFA (mg/day)**	0.498	< 0.001	0.539	< 0.001	0.554	< 0.001	0.409	< 0.001	0.491	< 0.001	0.277	< 0.001	0.442	< 0.001
**Iron (mg/day)**	0.221	< 0.001	0.120	0.001	0.164	0.004	0.136	< 0.001	0.162	< 0.001	0.352	< 0.001	0.298	< 0.001
**Magnesium (mg/day)**	0.354	< 0.001	0.364	< 0.001	0.402	< 0.001	0.271	< 0.001	0.333	< 0.001	0.303	< 0.001	0.421	< 0.001
**Zinc (mg/day)**	0.403	< 0.001	0.420	< 0.001	0.424	< 0.001	0.378	< 0.001	0.425	< 0.001	0.168	< 0.001	0.424	< 0.001
**Potassium (mg/day)**	0.352	< 0.001	0.356	< 0.001	0.406	< 0.001	0.258	< 0.001	0.322	< 0.001	0.116	0.001	0.320	< 0.001
**Calcium (mg/day)**	0.350	< 0.001	0.523	< 0.001	0.526	< 0.001	0.211	< 0.001	0.327	< 0.001	0.201	< 0.001	0.342	< 0.001
**Phosphorus (mg/day)**	0.455	< 0.001	0.505	< 0.001	0.525	< 0.001	0.355	< 0.001	0.439	< 0.001	0.260	< 0.001	0.458	< 0.001
**Vitamin B9 (μg/day)**	0.288	< 0.001	0.186	< 0.001	0.266	< 0.001	0.255	< 0.001	0.270	< 0.001	0.140	< 0.001	0.277	< 0.001
**Vitamin B12 (μg/day)**	0.446	< 0.001	0.539	< 0.001	0.524	< 0.001	0.447	< 0.001	0.505	< 0.001	0.200	< 0.001	0.505	< 0.001
**Vitamin A (RAE/day)**	0.178	< 0.001	0.139	< 0.001	0.211	< 0.001	0.102	0.004	0.137	< 0.001	0.083	0.02	0.143	< 0.001
**Vitamin D (μg/day)**	0.373	< 0.001	0.426	< 0.001	0.434	< 0.001	0.324	< 0.001	0.384	< 0.001	0.130	< 0.001	0.380	< 0.001
**Vitamin K (μg /day)**	0.287	< 0.001	0.198	< 0.001	0.272	< 0.001	0.240	< 0.001	0.263	< 0.001	0.113	0.002	0.265	< 0.001
**Vitamin E (mg/day)**	0.117	0.128	0.041	0.256	0.071	< 0.001	0.05	0.164	0.066	0.063	0.033	0.352	0.068	0.058

*BMI* body mass index; *CHO* carbohydrate; *SES* socioeconomic status; *SFA* saturated fatty acid

R denotes Pearson correlation coefficient

P value < 0.05 considered statistically significant

All the variables adjusted for HEI2015

## Discussion

To our knowledge, no other studies exist that investigate the relationship between dietary choline and betaine and risk of overweight/obesity and underweight/wasting in a sample of children in Iran. Our findings demonstrate that free choline, glycero-phospho-choline, phospho-choline, phosphatidyl choline, total choline, total betaine, and total betaine and choline were not related to overweight/obesity and underweight/wasting in Iranian six-year-old girls. However, we did observe total betaine intake was associated with mid-arm circumference.

Previous work exploring the association between dietary choline and betaine and anthropometric measures in children is largely inconsistent, and the relationship remains unclear. For example, betaine supplementation did not affect body composition in 42 obese adults following a hypo-energetic diet [50] or 42 untrained men [51]. Additionally, no significant difference in fat mass or fat-free mass was observed following six weeks of betaine supplementation in 29 trained CrossFit© athletes [52]. This was further supported by Long et al. who demonstrated, no significant differences in body weight or waist circumference across tertiles of participant betaine intake [53]. However, Cholewa et al. reported that betaine supplementation was related to improved body composition [30, 34]. On a similar note, no significant differences in anthropometric measures including weight, BMI, body fat percentage, waist circumference, hip circumference, and waist-to-hip ratio were observed in 96 diabetic patients following a two-month choline supplementation trial [31]. In contrast, a previous study reported improved body composition metrics following dietary choline and betaine supplementation, including total body fat percentage, trunk fat percentage, android fat percentage, gynoid fat percentage, body weight, BMI, waist circumference, and waist-to-hip ratio in 3214 participants across different age and sex categories [32].

These findings suggest that dietary choline and betaine may impact anthropometric measurements. Yet, the lack of consistent findings may be a reflection of several factors. First, different methods such as dual-energy x-ray absorptiometry (DXA), Bioelectric impedance analysis (BIA), BodPod, underwater weighing, and skin-fold calipers were used to assess anthropometric measurements in the literature. Although each measure has its own validity and reliability in context, the comparability of these measures should be considered. Second, men and women have different sex hormones, metabolites, and growth patterns that confound anthropometric measures, which were not considered in all studies. Lastly, to date, most of the literature has been conducted on adults, however, children engage in different dietary habits that influence their choline and betaine dietary intakes. As it stands, more research is required to expand our current understanding of the higher prevalence of obesity/overweight in girls and how dietary intake affects body composition in children.

Although choline and betaine’s mechanisms are not fully understood, a couple of theories have been suggested. Choline’s beneficial effect might be due to its capacity to reduce carnitine, which ultimately plays a role in fat mobilization and oxidation [54]. Further, choline, as a precursor for acetylcholine, may induce glucose uptake and lipolysis by activating m3 receptors in the brain [55]. However, due to the relationship between choline and other one-carbon metabolism components, the relationship is complex and still needs to be explored. Only one potential mechanism has been suggested for the relationship between betaine dietary intake and anthropometric measurements. It has been suggested that betaine can decrease acetyl-CoA carboxylase and lipoprotein lipase [34] and increase hormone-sensitive lipase and growth hormone [56].

Our study was the first to examine the association between different forms of dietary choline and betaine with anthropometric measurements among children. The cross-sectional design is the main limitation of the present study, as we are unable to determine causation. Although we controlled for multiple potential confounders, including SES and physical activity, our findings may be influenced by unknown residual confounders. Further, while FFQ is the best dietary assessment tool for large populations, recall bias could have influenced our results, and our study relied on self-reported measures by participant’s mothers. Finally, as our study sample was homogenous, we were unable to stratify our analysis based on sex or age.

## Conclusions

In conclusion, our cross-sectional study did not demonstrate an association between various forms of dietary choline intake and anthropometric measurements among six-year-old girls in Iran, but total betaine intake was associated with mid-arm circumference. Future prospective studies with a larger sample size in different populations are needed to delineate this relationship more clearly and explore potential mechanisms.

## Data Availability

The datasets used and/or analyzed during the current study are available from the corresponding author on reasonable request.

## Abbreviations

ANCOVA: Analysis of covariance
ANOVA: Analysis of variance
BIA: Bioelectric impedance analysis
BMI: Body mass index
CVD: Cardiovascular disease
DXA: Dual-energy x-ray absorptiometry
FFQ: Food frequency questionnaire
HC: Hip circumference
PA: Physical activity
SES: Socio-economic status
SFA: Saturated fatty acid
WHO: World Health Organization
WC: Waist circumference
WHR: Waist to hip ratio
